# P5C as an Interface of Proline Interconvertible Amino Acids and Its Role in Regulation of Cell Survival and Apoptosis

**DOI:** 10.3390/ijms222111763

**Published:** 2021-10-29

**Authors:** Magda Chalecka, Adam Kazberuk, Jerzy Palka, Arkadiusz Surazynski

**Affiliations:** Department of Medicinal Chemistry, Medical University of Bialystok, 15-089 Białystok, Poland; magda.chalecka@umb.edu.pl (M.C.); kadam568@gmail.com (A.K.); pal@umb.edu.pl (J.P.)

**Keywords:** Δ^1^-pyrroline-5-carboxylate, proline cycle, redox signaling, P5C synthase, P5C dehydrogenase, P5C reductase, PRODH/POX, ornithine aminotransferase, NADPH

## Abstract

Studies of cancer metabolism have focused on the production of energy and the interconversion of carbons between cell cycles. More recently, amino acid metabolism, especially non-essential amino acids (NEAAs), has been investigated, underlining their regulatory role. One of the important mediators in energy production and interconversion of carbons in the cell is Δ^1^-pyrroline-5-carboxylate (P5C)—the physiological intracellular intermediate of the interconversion of proline, ornithine, and glutamate. As a central component of these conversions, it links the tricarboxylic acid cycle (TCA), urea cycle (UC), and proline cycle (PC). P5C has a cyclic structure containing a tertiary nitrogen atom (N) and is in tautomeric equilibrium with the open-chain form of L-glutamate-*γ*-semialdehyde (GSAL). P5C is produced by P5C synthase (P5CS) from glutamate, and ornithine via ornithine *δ*-amino acid transferase (*δ*OAT). It can also be converted to glutamate by P5C dehydrogenase (P5CDH). P5C is both a direct precursor of proline and a product of its degradation. The conversion of P5C to proline is catalyzed by P5C reductase (PYCR), while proline to P5C by proline dehydrogenase/oxidase (PRODH/POX). P5C-proline-P5C interconversion forms a functional redox couple. Their transformations are accompanied by the transfer of a reducing-oxidizing potential, that affect the NADP+/NADPH ratio and a wide variety of processes, e.g., the synthesis of phosphoribosyl pyrophosphate (PRPP), and purine ribonucleotides, which are crucial for DNA synthesis. This review focuses on the metabolism of P5C in the cell as an interconversion mediator of proline, glutamate, and ornithine and its role in the regulation of survival and death with particular emphasis on the metabolic context.

## 1. Introduction

Recent studies on metabolism of cancer cells are focused on the role of proline, glutamate, and ornithine interconversions in regulation of redox potential for cell survival and death. An intermediate of these conversions is Δ^1^-pyrroline-5-carboxylate (P5C), which links the TCA cycle, urea cycle, and proline metabolism [1,2,3,4]. P5C has a cyclic structure containing a tertiary nitrogen atom (N) and is in tautomeric equilibrium with the open-chain form of L-glutamate-*γ*-semialdehyde (GSAL). It is produced by P5C synthase (P5CS) from glutamate [5] and ornithine via ornithine *δ*-amino acid transferase (*δ*OAT) [6,7]. P5C can be also converted to glutamate by P5C dehydrogenase (P5CDH) [8]. It is both a direct precursor of proline and a product of its degradation. The conversion of P5C to proline is catalyzed by P5C reductase (PYCR), while proline to P5C by proline dehydrogenase/oxidase (PRODH/POX) [6]. Initial studies on the role of P5C in cellular metabolism were focused on pathways that generate this intermediate in the conversion of proline, glutamate, and ornithine and their role in generating proline, a donor of the main substrate for collagen biosynthesis. In this system particularly important is PRODH/POX converting proline to P5C, during which ATP or ROS are generated, depending on the metabolic context. It has also been highlighted that conversion of P5C back into proline, catalyzed by P5C reductase (PYCR), is coupled to the pentose phosphate pathway, regenerating NADPH and producing nucleotides for DNA biosynthesis [9]. Thus, P5C participates not only in the regulation of survival processes—autophagy, growth, and proliferation, via involvement in the synthesis of biomass components but also in the regulation of cell death by apoptosis, due to the modulation of ROS levels dependent on the activity of (PRODH/POX).

## 2. Physicochemical Properties and P5C Levels in the Cell

Δ^1^-Pyrroline-5-carboxylate (P5C) has a cyclic structure with a tertiary nitrogen atom. It shows structural similarity to proline, however differs in the presence of a double bond in the ring [3]. In addition, P5C remains in tautomeric balance to the open-chain form of L-glutamate-*γ*-semialdehyde (GSAL) [1,10]. P5C is an unstable interconversion product of proline, ornithine, and glutamate. Recent studies have identified it as one of the most damage-prone endogenous metabolites [11]. These features of P5C have led to the challenge of measuring P5C levels in biological fluids for years. In 1984, a team of researchers developed an assay for P5C in biological fluids using PYCR purified from *Escherichia coli* [3]. The method allowed measurement of the fluctuations of P5C level in plasma and its concentration, which is in the lower micromolar range [1]. The study also proved that P5C can be derived not only from intracellular conversions of proline, ornithine, and glutamate but also can be an extracellular metabolite and depends on diet. What is especially interesting is that these fluctuations are independent of exogenous proline supplementation [12].

Currently, many studies confirm that P5C can regulate reducing-oxidizing potential and related processes [13,14,15,16,17]. The metabolic transformation of P5C is determined by the catalytic activity of enzymes that participate in its synthesis and utilization. Due to the diverse subcellular localization of these enzymes, important for P5C metabolism is its transport across the membranes of cellular organelles and intercellular transport. To investigate the mechanism of P5C transport, researchers used [U-^14^C]-L-pyrroline-5-carboxylate converted from [U-^14^C]-L-ornithine by partially purified rat liver OAT [18]. It has been demonstrated that P5C transport is different from other amino acid transport and depends on ATP, sodium ions, and transporter that is in close relationship with PYCR [19,20]. Confirmation of this hypothesis was found in the results of study of structural analogues of both P5C and its chain form—GSAL, which were designed to inhibit P5C uptake.

The involvement of amino acid transport systems—system A, ASC, L in P5C transport was excluded [20]. The studies provided evidence for the existence of a carrier protein for P5C in the membrane [19,20]. Although P5C transport exhibits saturation kinetics and energy dependence, P5C uptake does not form a concentration gradient. On the opposite, cells incubated with radiolabelled P5C over a wide range of experimental conditions did not accumulate P5C but completely converted P5C to proline. This led to the suspicion that its carrier is closely related to its conversion to proline. Thus, it was postulated that it might be related to PYCR activity [20]. In the study, structural analogues of P5C inhibited its re-uptake [19]. Also, it was shown that all amounts of P5C were recovered as proline under inhibited P5C uptake. Therefore, the mechanism of inhibition did not directly involve PYCR activity. Studies on a number of cell lines have suggested that the carrier protein is associated, at least functionally, with P5C reductase, which converts P5C to proline. Specific inhibitors of P5C uptake would help elucidate this mechanism [19]. Moreover, the suppressive effect of these inhibitors on the oxidative potential transfer, which is mediated by P5C was proven, as the analogues used inhibited the pentose phosphate pathway (PPP) and consequently the synthesis of DNA [19].

As a cyclic proton acceptor, P5C can act as a messenger both inside the cell and between cells. It has been shown that P5C can be released from one tissue and via the extracellular fluid, can impact the function of other tissues [1].

## 3. The Metabolic Pathways of P5C

Initial studies on the role of P5C in cellular metabolism were focused on pathways that generate this intermediate in the conversion of proline, glutamate, and ornithine and their function in generating proline, a donor of the main substrate for collagen biosynthesis [4,21]. Currently, it is recognized that P5C as a product of proline, glutamate, and ornithine interconversion is involved in the regulation of cell growth, redox balance, production of ATP, and immunomodulation [1,22,23]. It has been demonstrated that inability to generate P5C in the cell, leads to proline auxotrophy. However, if one of the pathways for P5C synthesis (from ornithine or glutamate) is present, that normal cell growth is assured, despite the deficiency of proline in the medium [1,24].

**Figure 1:**
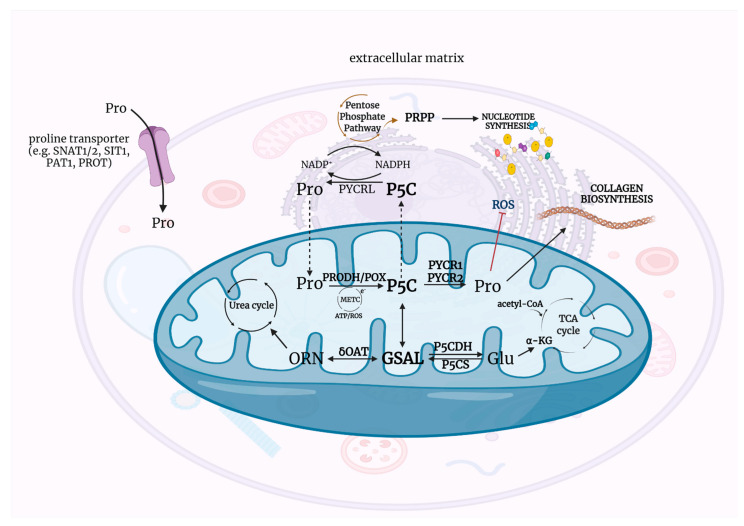
The metabolic pathways of P5C—physiological intracellular intermediate of the interconversion of proline, ornithine, and glutamate. Acetyl-CoA—acetyl coenzyme A, ATP—adenosine triphosphate, α-KG—α-ketoglutarate, Glu—glutamate, GSAL—L-glutamate-*γ*-semialdehyde, METC—mitochondrial electron transport chain, NAD^+^—oxidized form of nicotinamide adenine dinucleotide, NADH—reduced form of nicotinamide adenine dinucleotide, NADP^+^ –oxidized form of nicotinamide adenine dinucleotide phosphate, NADPH—reduced form of nicotinamide adenine dinucleotide phosphate, ORN—ornithine, P5C—Δ^1^-pyrroline-5-carboxylate, P5CDH—P5C dehydrogenase, P5CS—P5C synthase, PAT1—proton-coupled amino acid transporter 1, PRODH/POX—proline dehydrogenase/oxidase, PROT—L-proline transporter PROT, PYCR1/2/L—P5C reductase 1/2/L, ROS—reactive oxygen species, SIT1—sodium/imino-acid transporter 1, SNAT1/2—sodium-coupled neutral amino acid transporter 1/2, TCA cycle—tricarboxylic acid cycle, *δ*OAT—ornithine δ-aminotransferase. Created with BioRender.com (accessed on 26 October 2021).

Recently, P5C has been of great interest especially in the context of regulation of reducing-oxidizing potential in the cell. One of the first reports confirming the involvement of P5C in the regulation of redox potential dates back to 1985 [1]. At that time, studies on rats provided evidence that food deprivation impaired rate of collagen biosynthesis in connective tissues independently of hydroxylation of proline but depended only on energy metabolism and the availability of proline—the main substrate for collagen production [21,25]. These observations became the basis for an attempt to understand the precise mechanisms that determine the contribution of proline-P5C to the regulation of the cellular redox potential and its metabolic implications. P5C with proline is a redox couple and provides transfer of reducing potential from the cytosol to the mitochondria and oxidative potential from the mitochondria to the cytosol [26,27]. Thus, acting as redox potential carriers, they regulate metabolic pathways that depend on reducing-oxidizing levels in the cell: STAT3-MAPK/NF-kB [28], Jun N-terminal kinase (JNK) [15,29], and AKT and mTOR [30]. However, the metabolism of P5C and proline would not be possible without the availability of a group of specific enzymes, that together with P5C and proline, establish the P5C-proline cycle. Figure 1 presents the Δ^1^-pyrroline-5-carboxylate (P5C) metabolic pathway in the context of proline, ornithine, and glutamate interconversion.

## 4. Enzymes of the P5C Metabolism

### 4.1. P5C Synthase

P5C synthase (P5CS) is a mitochondrial enzyme, which activity is dependent on the presence of ATP and NAD(P)H [5,31]. P5CS is a multifunctional enzyme—containing two enzymatic domains in its structure, glutamate 5-kinase (G5K; EC 2.7.2.11) and γ-glutamyl phosphate reductase (γ-GPR; EC 1.2.1.41), and therefore P5CS catalyzes the coupled conversion of phosphorylation and reduction of glutamate to P5C [6,32]. However, in bacteria and lower eukaryotes, the two domains are separate enzymes: G5K and γ-GPR [7,32]. In the first step of P5C synthesis, glutamate 5-kinase with the participation of adenosine triphosphate (ATP) activates the gamma carbon of glutamate and catalyzes its phosphorylation. This results in the formation of an unstable reactive intermediate product—G5K [33], which in the second step, is reduced to L-glutamate-*γ*-semialdehyde (GSAL) in an NADPH-dependent reaction. GSAL is in tautomeric equilibrium with P5C, undergoing spontaneous non-enzymatic cyclization to P5C with a loss of water [5,32].

Previous studies have shown, that human P5CS undergoes alternative splicing to form two mature RNA transcripts which encode two isoforms, long (P5CS.long) and short (P5CS.short). These isoforms differ by two amino acids at the N-terminal of the active center in glutamate 5-kinase, which results in different sensitivity to their inhibition by ornithine [5,34] however removal of ornithine from the environment, restores P5C synthase activity (P5CS).

P5CS.short is localized in the intestine, is inhibited by ornithine, and is required for arginine synthesis. In contrast, P5CS.long is found in most tissues, is involved in the conversion of glutamate to proline, and has no sensitivity to ornithine. It is also known, that P5CS.long is hormonally regulated: up-regulated by estradiol and down-regulated by hydrocortisone and dexamethasone [5]. In addition, studies have shown that in colon cancer cells DLD-1, P5CS.long is up-regulated by p53—a tumor suppressor, that plays a key role in cell cycle regulation, angiogenesis, differentiation, and activation of apoptosis [5]. P5CS knockdown has been shown to impair cell growth and proliferation, due to ornithine and arginine deficiency [35]. However, the primary function of P5CS is the production of P5C, which is converted to proline by the P5C reductase (PYCR) [36].

### 4.2. Ornithine Aminotransferase

The important source of P5C in the cell (in addition to proline and glutamate) is ornithine. This amino acid is converted to P5C by a reversible reaction catalyzed by ornithine *δ*-amino acid transferase (OAT; EC 2.6.1.13). OAT, like P5CS, is a mitochondrial enzyme [37]. Reversible conversions of P5C and ornithine, allow transfer of carbons between the TCA cycle and urea cycles, which are crucial for cell function [2,4,38]. OAT is considered to be the enzyme that catalyzes both the conversion of ornithine to P5C and P5C to ornithine, however, deficiency of this enzyme results in the accumulation of ornithine in the cell, which directly affects the activity of P5CS and leads to loss of both substrates for proline biosynthesis [39]. In addition, studies have shown that the glutamate-P5C-ornithine pathway is used in animals, also in humans, to produce ornithine in erythrocytes, for the maintenance of proper homeostasis of the urea cycle and synthesis of citrulline and arginine, especially in the case of deficiencies of exogenous ornithine [40].

An unusually important function of OAT activity is the fact that it provides P5C as a substrate for PYCRL, the cytoplasmic isoform of P5C reductase. PYCRL uses NADP^+^ as a co-factor, which is utilized by the oxidative arm of the pentose phosphate pathway to synthesize nucleotides and new DNA [41,42].

### 4.3. Proline Dehydrogenase/Oxidase

The transformation of P5C in the cell is closely related to proline, which is both the product of its degradation and the substrate for its production. The synthesis of P5C from proline is catalyzed by proline dehydrogenase/oxidase, also known as proline dehydrogenase (PRODH/POX; EC 1.5.5.2), a flavin-containing enzyme that couples the oxidation of proline with reduction of membrane-bound ubiquinone or coenzyme Q. PRODH/POX is located in the mitochondrial inner membrane [6]. The conversion of proline to P5C is linked to cytochrome c and FAD as electron and hydrogen acceptors in the respiratory chain [43]. Thus, the electron pair given by proline can impact the phosphorylation of two ADP molecules and the formation of two ATP molecules [40]. The oxidation of proline to P5C is accompanied by the generation of reactive oxygen species (ROS), which by increasing oxidative stress can contribute to the induction of apoptosis [44]. PRODH/POX initiates both intrinsic and extrinsic apoptotic pathways, possibly through NFAT and MEK/ERK signaling [6,45,46]. The gene encoding PRODH/POX, known as PRODH, is under the transcriptional control of p53 (a main regulator of apoptosis) [47,48,49] and peroxisome proliferator-activated receptor gamma (PPARγ) [50,51,52,53]. In addition, to induce apoptotic cell death, PRODH/POX inhibits tumor progression by down-regulating growth and differentiation of tumor cells, by inhibiting the cell cycle at the G2-M checkpoint [54]. On the other hand, regulation of PRODH/POX activity plays a particular role in the redox signaling system for apoptosis, and in different metabolic contexts, for autophagy or survival [55].

### 4.4. P5C Reductase

The conversion of P5C to proline is mediated by P5C reductase (PYCR), accompanied by the oxidation of NADPH to NADP^+^ [30,56]. Three PYCR isoforms have been identified in the human body, which are products of three homologous genes: PYCR1 (17q25.3), PYCR2 (1q42.13), and PYCRL (8q24.3.7) [30]. PYCR1 and PYCR2 show 84% structural homology and are located in the mitochondrial intermembrane space and mitochondrial matrix, respectively [57]. They are mainly involved in the conversion of P5C to proline, in the glutamine-P5C-proline pathway. In contrast, PYCRL, unlike PYCR1 and PYCR2, is 40 amino acids shorter at the C-terminus. It is located in the cytosol and is involved in the conversion of P5C to proline; however, the source of P5C is ornithine [58,59]. The preferred co-factor for mitochondrial isoforms is NADH, while for cytosolic isoforms it is NADPH [58] in interlock to G6PD and 6PGD of the pentose phosphate pathway [9] which is important for purine nucleotide synthesis [42,60].

In many cancer cell types, there is a marked increase in PYCR expression, which is correlated with poor prognosis. The consequence of PYCR overexpression is high levels of proline in the cell, whose metabolism is part of the metabolic reprogramming of the tumor [16,54,61]. An increase in activity of P5C reductase, contributes to the increase in intracellular proline, a substrate for PRODH/POX, resulting in an increase in ATP synthesis in the cell. This determines cell proliferation and inhibition of apoptosis induction via cell cycle regulation, redox homeostasis, and promotion of signaling pathways, such as STAT3-MAPK/NF-kB [28], Jun N-terminal kinase (JNK) [15], and AKT and mTOR [30]. Studies have shown that PYCR1 is responsible for maintaining PRODH/POX activity during proline deficiency [13]. Furthermore, it was shown that as a consequence of PYCR1 knockdown, there was significant activation of apoptosis and inhibition of proliferation through the inactivation of signaling pathways of survival processes [62,63]. On the other hand, silencing of the gene encoding PYCR1 inhibited the cell cycle in the S/G1 phase by down-regulating cyclin D1 [63] and in the G2/M phase by reducing the expression of CDK1, CDK2, CDK4, and Cyclin B1 [64]. In contrast, silencing of PYCR2 resulted in decreased proliferative capacity and activation of AMPK/mTOR-induced autophagy in melanoma cells [65]. Reduction of PYCRL expression due to severe proline deficiency in the cell markedly inhibited cell proliferation [66].

A significant role of PYCR has been assigned in regulating redox homeostasis and ROS levels mainly through proline biogenesis and balancing the NADP^+^/NADPH ratio [59]. As a component of the antioxidant system, proline could also protect cells from oxidative stress [67].

### 4.5. P5C Dehydrogenase

The role of P5C dehydrogenase (P5CDH) is the NAD^+^-dependent conversion of P5C to glutamate [8,22]. The enzyme is ubiquitous, however, it shows differences in activity depending on the tissue. It is usually associated with high PRODH/POX expression [1]. P5C dehydrogenase is mainly localized in the mitochondria, although it is also found in the cytosol [68]. Glutamate, as a product of P5C dehydrogenase activity, is further converted to α-ketoglutarate (α-KG), and this process transfers carbon to the tricarboxylic acid cycle (TCA) and ammonia to nitrogen recycling [69,70,71]. In addition, α-KG is not only a major substrate for the TCA cycle but also an important factor in the prolyl hydroxylation of HIF-1α. Thus, the changes in α-KG availability regulate HIF-1α stabilization and the response to hypoxia [72]. It is also known that glutamate is involved in the synthesis of glutathione, which is an important factor in protection against oxidative stress in the cell [73].

## 5. Biological Role of P5C

Δ^1^-Pyrroline-5-carboxylate (P5C) demonstrates a pleiotropic role not only as a redox couple with proline or as a transporter of redox equivalents between the mitochondria and the cytosol via PRODH/POX and PYCR. P5C is a common element in the interconversion of the triad of amino acids: proline, glutamate, and ornithine, becoming a central metabolic component that maintains cellular homeostasis. In addition to providing C and N for the formation of essential components of cellular biomass—amino acids, nucleotides, and hexosamines—it serves as a fuel for the TCA cycle and indirectly plays an important role in gluconeogenesis. Thus, it has been postulated to function as a regulatory factor in cell growth, cell death, ATP production, and immunomodulation [22,23].

### 5.1. The Importance of the P5C-Proline Cycle in Cancer Metabolism

**Figure 2:**
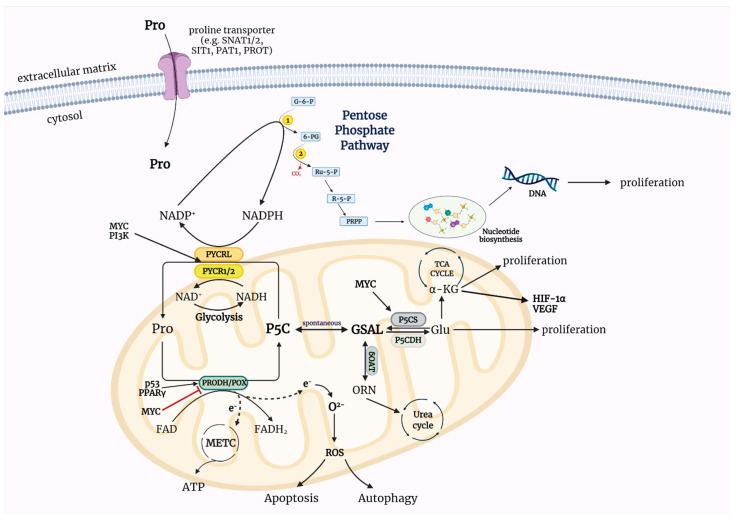
The importance of the P5C-proline cycle in cancer metabolism. Metabolism of P5C depended on specific enzymes establish to the P5C-proline cycle. The activity of these enzymes is accompanied by transport of redox equivalents between the mitochondria and the cytosol that determine cellular processes—proliferation, survival, apoptosis, autophagy. The interconversion of P5C and proline changes the NADP^+^/NADPH ratio in the cell leading to regulation of phosphoribosyl pyrophosphate (PRPP) synthesis and nucleotide synthesis via pentose phosphate pathway. ATP—adenosine triphosphate, FAD—oxidized form of flavin adenine dinucleotide, FADH_2_—reduced form of flavin adenine dinucleotide, Glu—glutamate, GSAL—L-glutamate-*γ*-semialdehyde, HIF-1α—hypoxia-inducible factor 1, METC—mitochondrial electron transport chain, MYC—myelocytomatosis oncogene cellular homologue, NAD^+^—oxidized form of nicotinamide adenine dinucleotide, NADH—reduced form of nicotinamide adenine dinucleotide, NADP^+^—oxidized form of nicotinamide adenine dinucleotide phosphate, NADPH—reduced form of nicotinamide adenine dinucleotide phosphate, ORN—ornithine, oxPPP—oxidative arm of the pentose phosphate pathway, p53—tumor protein 53, P5C—Δ^1^-pyrroline-5-carboxylate, P5CDH—P5C dehydrogenase, P5CS—P5C synthase, PAT1—proton-coupled amino acid transporter 1, PI3K—phosphoinositide 3-kinase, PRODH/POX—proline dehydrogenase/oxidase, PROT—L-proline transporter PROT, PYCR1/2/L—P5C reductase 1/2/L, PPARγ—peroxisome proliferator-activated receptor gamma, ROS—reactive oxygen species, SIT1—sodium/imino-acid transporter 1, SNAT1/2—sodium-coupled neutral amino acid transporter 1/2, TCA cycle—tricarboxylic acid cycle, VEGF—vascular endothelial growth factor, α-KG—α-ketoglutarate, *δ*OAT—ornithine δ-aminotransferase, G-6-P—glucose-6-phosphate, 6-PG—6-phosphogluconate, Ru-5-P—ribulose-5-phosphate, R-5-P—ribose-5-phosphate, PRPP—phosphoribosyl pyrophosphate, 1—glucose-6-phosphate dehydrogenase (G6PDH), 2—6-phosphogluconate dehydrogenase (6PGD). Created with BioRender.com (accessed on 26 October 2021).

Figure 2 demonstrates biological role of the P5C-proline cycle in cancer cell metabolism. P5C metabolism is crucial for cancer cells because, unlike glucose, it is not dependent on oxygen. In cancer cells, glucose is used for energy production (Warburg effect) [74,75], while P5C is the main anaplerotic substrate, leading to the replenishment of the TCA cycle intermediates so that the Krebs cycle does not stop.

Moreover, the metabolism of P5C is associated with proline biosynthesis, and in particular, the utilization of glutamine-derived carbon for proline and collagen synthesis is of significant interest. It is suggested that metabolism of proline and hydroxyproline (HyPro), as products of collagen degradation, is an alternative to canonical bioenergetic and signaling pathways under hypoxic and starvation conditions in cancer cells [55,76]. This is attributed to the coupling of the redox cycle interlocking with PPP to the proline cycle that generates ATP and NADP^+^ in mitochondria, termed the “Proline shuttle” (PS). It has been found that the reducing potential from glucose in the form of NADPH affects ATP levels through the proline cycle [2]. Phang et al. first showed that NADPH could fuel mammalian respiratory activity without stoichiometric consumption of proline [77]. This served to describe the proline cycle [78]. It is now known that the P5C-proline cycle enhances oxPPP, maintains cytosolic levels of pyrimidine nucleotides, and generates reactive oxygen species (ROS) to activate signaling pathways in cells [22,36,77]. The interconversion of P5C and proline is accompanied by the transfer of a reducing-oxidizing potential that changes the NADP^+^/NADPH ratio in the cell, resulting in the regulation of particular transformations, such as the synthesis of phosphoribosyl pyrophosphate (PRPP) or purine ribonucleotides [40]. This aspect of P5C-mediated regulation of redox potential appears to be crucial in the context of modulation of cellular processes determining cell survival and cell death.

Proline has been implicated as a potent sensor of oxidative stress, showing the ability to protect cells from ROS derived from different sources, e.g., H_2_O_2_. In bacteria, proline plays a protective role against osmotic stress, allowing bacteria to grow in saline environments. On the other hand, in plants, increased proline levels are a response to water deprivation, salt stress, and low temperature [21,40]. The P5C-proline cycle, through regeneration of oxidative potential, directs glucose and glutamate carbons to produce cellular biomass that is essential for growth and proliferation. This is particularly important in the nature of cancer because cancer cells have a significant requirement for the building molecules—nucleotides, amino acids, and fatty acids [79].

A characteristic feature of cancer is an unusually dynamic metabolism, demonstrated by uncontrolled processes of growth, proliferation, metastasis formation, and impaired activation of cell death under stressful conditions [75]. Metabolic reprogramming causes cancer cells to adapt to adverse conditions, especially oxygen deprivation. A specific mechanism exploited by cancer cells is the generation of NAD^+^, a co-factor essential for the glycolytic pathway at the glyceraldehyde-3-phosphate dehydrogenase step [80]. NAD^+^ generation accompanies the conversion of pyruvate to lactate. Thus, to maintain the activity of the glycolytic pathway, the cancer cell dedicates pyruvate to the synthesis of lactate, rather than to the production of acetyl-CoA, which is not only a substrate for fatty acid synthesis but also an essential component of the TCA cycle. Similarly, an important aspect in the production of cellular biomass necessary for proliferation is the generation of NADP^+^, which is a co-factor involved in ribose synthesis via the oxidative arm of the pentose phosphate pathway (oxPPP). Studies have shown that NADP^+^ is required for the first two steps of ribose synthesis which include glucose-6-phosphate dehydrogenase (G6PDH) and 6-phosphogluconate dehydrogenase (6PGD) activities [81]. Knockdown of enzymes involved in proline and glutamine interconversion inhibit the cancer cell growth rate, suggesting a role for P5C in regulation of redox potential as a factor that determines cell growth and proliferation. In an experiment on lung cancer cells (with high MYC expression), the effects of MYC knockdown and the enzymes under the control of MYC, PYCR1/2/L, and P5CS, were investigated [10,82]. This study demonstrated that the knockdown resulted in a decreased proliferation rate, whereby the addition of P5C compensated to some degree for all enzymes, except PYCRL. Knockdown of P5CS inhibited the glutamate-P5C-proline pathway, but the addition of P5C and proline reversed the effect of P5CS knockdown. Furthermore, the effect of simultaneous knockdown of P5CS and PRODH/POX was investigated and it was shown that the proliferation rate was markedly decreased, but only proline reversed this effect slightly. Knockdown of P5CS was demonstrated to cause an overall decrease in NADP^+^ and NAD^+^ levels. A similar effect was obtained in response to the knockdown of all PYCR isoforms simultaneously. However, in this case, neither the addition of proline orP5C reversed this effect [22].

### 5.2. Collagen as a Source of Proline

Collagen, the most abundant protein in mammals is represented by 29 genetically distinct types of proteins [83]. The most abundant are collagen type I–IV. Their characteristic feature is the ability to create a trihelical structure composed of identical or different alpha subunits [84] composed of repeating amino acid triplets containing glycine and often proline and hydroxyproline. The hydroxyproline is formed by hydroxylation of prolyl residues in the newly synthesized collagen by prolyl hydroxylase [85]. It is the critical process in collagen biosynthesis. The presence of proline and hydroxyproline, constituting about 25% of all collagen amino acids allows the formation of a triple helix. The trihelical structure allows for the secretion of the molecule outside the cell and makes it resistant to the action of non-specific proteases [86]. Extracellular collagen is degraded by specific metalloproteinases that cut the molecule into two parts that are further degraded by non-specific proteases. The resulting short fragments are internalized and degraded intracellularly in the lysosomes to free amino acids, with the exception of imido-dipeptides, e.g., glycyl-proline. Imido-dipeptides are degraded by the cytoplasmic prolidase [87] (Figure 1). It is estimated that in this way the cell recovers about 90% of proline, which can be used for the biosynthesis of new proteins, including collagen [88,89]. However, during the last few decades it has been recognized that proline could play several other functions, e.g., as a stress molecule, regulator of transcription factors and substrate for proline dehydrogenase/proline oxidase (PRODH/POX)-dependent apoptosis/autophagy [38,90]. Particular attention is focused on the role of proline availability in driving PRODH/POX-dependent functions. In this context, the activity of prolidase (proline supporting enzyme) and collagen biosynthesis (proline utilizing process) could be of critical importance in providing proline for PRODH/POX-induced apoptosis, autophagy or survival. Proline released from collagen degradation can be a source of energy and is linked to redox signaling, regulation of protein synthesis, cancer cell proliferation and metabolism. The role of proline availability in regulation of mTOR and AAR-ATF4 pathways and cancer cell growth has been well established [91]. An interesting interplay between proline metabolism/collagen synthesis and microenvironment stress was reported in a recent study on lung cancer, in which the authors show the interaction of PYCR1 in the mitochondria with Kindlin-2, a protein critical for integrin-mediated cell-ECM adhesion. ECM stiffness characteristic for cancer ECM induces kindlin-2 translocation into mitochondria where it interacts with PYCR1, increasing PYCR1 activity and subsequently proline level. In vivo kindlin-2 ablation strongly reduced PYCR1 and proline level, fibrosis, tumor growth and mortality rate [91,92]. Since PYCR1 is overexpressed in breast cancer, prostate cancer, and lung cancer cells [63,93,94], the silencing of PYCR1 expression could represent approach for cancer treatment [63,93,95]. However, specific inhibitors of PYCR1 are not available so far. On the other hand, collagen synthesis is upregulated under conditions of excess of reducing potential. In these conditions the incorporation of proline into collagen removes it from the metabolic pool. Thus, by using collagen as a waste disposal process for reducing equivalents in the form of proline, tumors can optimize conditions for metabolism and growth. The above data suggest that collagen metabolism (synthesis and degradation) through modulation of proline availability is involved in regulation of complex cellular metabolism.

### 5.3. The Role of Amino Acid Transporters in Proline Traffic

Specific membrane transport systems play a supportive role in providing proline for cancer cells. The SLC family of tissue-specific transporters provide proline for protein synthesis, regulation of cell signalling and metabolism, maintenance of redox balance and adaptation to osmotic stress. The high affinity of SLC transporters ensures uptake of proline and utilization of this amino acid as a source of carbon, nitrogen, and energy. They transport proline in a pH gradient-dependent, proton-coupled manner e.g., PAT1 or sodium-coupled neutral amino acid transport type A, (SNAT1 and SNAT2) and the sodium/imino-acid transporter (SIT1) [96] (Figure 1 and Figure 2). Their significance in cell differentiation and proliferation of embryonic stem (EC) cells has been demonstrated [97]. Of interest are also studies of Kudo et al. showing that in human osteoblast-like SaOS-2 cells proline uptake through the transport system A, (SNAT1 and SNAT2) is stimulated by insulin-like growth factor-I (IGF-I). This is particularly important in the context of proline and collagen metabolism since IGF-I is involved in proline-dependent stimulation of collagen expression and biosynthesis [98,99].

Kinetic analysis showed that IGF-I enhanced proline transport by increasing the maximum velocity (V_max_) without significant changes in the affinity (Michaelis constant, Km) of the carrier for the substrate. This experiment demonstrated that Na^+^-dependent proline uptake was also stimulated by insulin-like growth factor-II and insulin-like growth factor-I analogues [97]. Proline transport could be of great importance in the central nervous system. A representative of this group of transporters is PROT (SLC6A7), which is brain-specific and located in glutamatergic neurons with partial apposition to AMPA and NMDA receptors [96,100,101,102]. Furthermore, members of the amino acid A transport system, SNAT1 (SLC38A1) and SNAT2 (SLC38A2), are expressed in both glutamatergic neurons and glial cells and regulate the intracellular proline pool. It has been found that proline transport in C6 glioblastoma cells occurs mainly through a saturable Na^+^-dependent mechanism and the uptake process can be distinguished into two components, system A and system ASC [103]. Most of the transport is performed by system A in response to amino acid deprivation. It has been documented that the transport system is stereospecific, and can be inhibited by proline derivatives such as methyl and benzyl esters, hydroxyproline as well as absence of Ca^2+^, while in the presence of Ca^2+^ the proline transport is significantly stimulated [103]. Although the proline transport system could be of critical importance in the central nervous system, in peripheral tissues it has a complementary function. Intracellularly, proline is synthesized from glutamine or ornithine. It has been found that in fibroblasts glutamine deprivation contributed to relative proline deficiency. In such a case glutamine shortage favors transport of exogenous proline as a substrate for intracellular pathways [98]. At least in respect to collagen biosynthesis, exogenous proline has lower than glutamine influence on the process [104]. It seems that proline transporters are only single players in complex regulatory machinery of cellular metabolism that determine the source of proline availability for cell metabolism.

### 5.4. Involvement of P5C in the Activation of the Pentose Phosphate Pathway

Regulation of the oxidative arm of the pentose phosphate pathway (oxPPP) is one of the primary mechanisms for counteracting oxidative stress. It leads to the production of NADPH, which is essential for glutathione reduction, redox transfer, and fatty acid synthesis. In addition, oxPPP provides PRPP for nucleotide synthesis [105]. It has been emphasized that an increase in the flux of oxPPP accompanies the activation of T lymphocytes [35] and it has been suggested that the ratio of the contribution of the oxidative and non-oxidative arm of PPP (non-oxPPP) to the formation of PRPP, depends on the redox balance. Already by the 1970s it had been demonstrated that the NADP+/NADPH ratio is a factor which determines the rate of PRPP formation [81]. It is also known that p53 through the regulation of PPP is involved in promoting NADPH production [106], whereas activation of the MYC oncogene impairs the protection mechanism against oxidative stress, which is consistent with the NADP^+^/NADPH shift during proliferation [107]. When NADH is in excess, NADPH can be formed from NADP^+^ in mitochondria via NADP^+^ transhydrogenase [108]. P5C plays a key role in the regulation of oxPPP.

Using a method to produce ^14^CO_2_ from glucose-1-^14^C it was shown that P5C added to cell cultures markedly enhanced oxPPP activity [1,41]. Moreover, metabolic interlock withG6PDH of the PPP was demonstrated in experiments on human erythrocytes, which have high PYCR expression but are deprived of PRODH/POX activity [9]. Redox transfer significantly enhanced oxPPP and increased the formation of PRPP, a key compound for both de novo DNA synthesis and the salvage pathways of nucleic acid synthesis [42,60]. In studies, it has been observed that treatment of human erythrocytes with P5C, increases the level of ribosyl-5-phosphate (R5P) and then incorporates the formed purines into nucleotides in the rescue pathway in the PPRP-dependent process [9], and the addition of P5C to red blood cells greatly increases the incorporation of labeled purines into the appropriate nucleotides [1,42,60,109]. Other research has shown that knockdown of P5CS has impaired oxPPP activity, and treatment of P5C compensated for this effect. In contrast, the knockdown of PYCR1, PYCR2, and PYCRL causes decreased oxPPP activity and the addition of P5C did not reverse this phenomenon. Furthermore, it was observed that the knockdown of PYCR reductase reduced NAD^+^ and NADP^+^ levels and inhibited glycolysis and cell proliferation [36]. oxPPP is stimulated by oncogenes, although there are other pathways e.g., non-oxidative arms of PPP that are preferred by cancer cells to produce ribose, depending on the metabolic context—especially on glucose availability [110]. During the activation phase of proliferative processes, oxPPP is activated by G6PD and P6GD. Additional stimulation of PYCR and generation of NADP^+^ causes oxPPP to proceed rapidly and efficiently, with the production of R5P and PRPP, which is required for the synthesis of pyridine nucleotides and purine and pyrimidine nucleotides [1,111]. Experiments on lung cancer cell lines have shown that the addition of P5C and increased expression of MYC through up-regulation of the enzymes P5CS, PYCR1, PYCR2, PYCRL increases glycolysis. In contrast, the knockdown of P5CS and PYCR causes a decrease in glycolysis rate, ATP production, and oxPPP activity [36].

### 5.5. Interconversions of Proline, Glutamate, and Ornithine in the Context of Regulation of Redox Transfer

The main role attributed to P5C is to maintain hypoxic homeostasis through the “Proline shuttle” and to be an intermediate in the interconversions of proline, glutamate, and ornithine, which aim to optimize metabolic pathways in the cell [1]. Although the aforementioned amino acids are classified as non-essential amino acids (NEAA) and are not essential in the diet, in fact, their conversions have key functions in the regulation of cellular processes related to the proper functioning of the TCA cycle and the provision of glucose-independent energy to the cell [112,113]. Through the mutual conversions of proline, glutamate, and ornithine, the cell is able to compensate for their deficiencies and regulate its levels. For instance, high levels of ornithine in cells decrease P5CS activity and promote P5C synthesis from ornithine. Decreasing ornithine levels activate P5CS and restore P5C synthesis from glutamate, and further downstream P5C is converted to proline by PYCR. This reaction undergoes negative feedback and when there is an excess of proline in the cell, P5CS is inhibited.

An important aspect of the conversion of proline, ornithine, and glutamate is their effect on the NADP^+^/NADPH ratio in the cell. Conversion of ornithine to proline is accompanied by a transfer of the reduction potential from cytosolic NADPH to mitochondrial NADP^+^. In contrast, in the conversion of glutamate to proline, two NADPH molecules are oxidized—one in the cytosol and one in the mitochondria. Likewise, the conversion of glutamate to ornithine is accompanied by a potential transfer from NADPH to NADP^+^.

On the other hand, the conversion of proline to glutamate involves two steps, during which two molecules of ATP are formed as a result of PRODH/POX activity (proline →P5C), whereas NADH is produced via P5C dehydrogenase (P5C→glutamate). The generated glutamate can become a precursor of α-ketoglutarate (α-KG), a substrate for the TCA cycle crucial for the bioenergetics of the cell.

The described interconversions of proline, ornithine, and glutamate cause the transfer of redox potential between different cell compartments, transferring electrons from NADH to NAD^+^, which is a co-factor necessary for the glycolytic pathway (Figure 2), thus this confirms the key involvement of these transformations not only in maintaining the NADP^+^/NADPH balance but also NAD^+^/NADH.

## 6. Conclusions

Proline, glutamate, and ornithine are substrates for the production of Δ^1^-pyrroline-5-carboxylate (P5C) that links the TCA cycle, urea cycle, and proline metabolism. In this system, particularly important is PRODH/POX converting proline to P5C, during which ATP and ROS are generated, depending on the metabolic context. It has been also highlighted that conversion of P5C back into proline, catalyzed by P5C reductase (PYCR), is coupled to the PPP regenerating NADPH and producing nucleotides for DNA biosynthesis. Interconversion of proline, glutamate, and ornithine with P5C as an intermediate, play a pivotal role in regulating many processes that depend on redox potential for cell survival and death.

Further exploration of their biochemical aspects will be essential for a better understanding of cancer cell metabolism and the possibility of developing effective therapies.

## Abbreviations

6-PG	6-phosphogluconate
6PGD	6-phosphogluconate dehydrogenase
AAR	Amino acid response
acetyl-CoA	Acetyl coenzyme A
ADP	Adenosine diphosphate
AKT	Protein kinase B
AMPA	α-amino-3-hydroxy-5-methyl-4-isoxazolepropionic acid receptor
AMPK	AMP-activated protein kinase
ATF4	Activating transcription factor 4
ATP	Adenosine triphosphate
Ca^2+^	Calcium ion
CDK	Cyclin-dependent kinase
DNA	Deoxyribonucleic acid
ECM	*Extracellular matrix*
ERK	Extracellular signal-regulated kinases
FAD	Oxidized form of flavin adenine dinucleotide
FADH_2_	Reduced form of flavin adenine dinucleotide
G5K	Glutamate 5-kinase
G-6-P	Glucose-6-phosphate
G6PDH	Glucose-6-phosphate dehydrogenase
Glu	Glutamate
GSAL	L-glutamate-*γ*-semialdehyde
H_2_O_2_	Hydrogen peroxide
HIF-1α	Hypoxia-inducible factor 1α
IGF-I	Insulin-like growth factor-I
JNK	Jun N-terminal kinase
Km	Michaelis constant
MAPK	Mitogen-activated protein kinase
METC	Mitochondrial electron transport chain
mTOR	Mechanistic target of rapamycin
MYC	Myelocytomatosis oncogene cellular homologue
NAD^+^	Oxidized form of nicotinamide adenine dinucleotide
NADP^+^	Oxidized form of nicotinamide adenine dinucleotide phosphate
NADH	Reduced form of nicotinamide adenine dinucleotide
NADPH	Reduced form of nicotinamide adenine dinucleotide phosphate
NEAA	Non-essential amino acid
NFAT	Nuclear factor of activated T-cells
NF-kB	Nuclear factor kappa-light-chain-enhancer of activated B cells
NMDA	N-methyl-D-aspartate receptor
non-oxPP	Non-oxidative arm of the pentose phosphate pathway
ORN	Ornithine
oxPPP	Oxidative arm of the pentose phosphate pathway
p53	Tumor protein 53
P5C	Δ^1^-pyrroline-5-carboxylate
P5CDH	P5C dehydrogenase
P5CS	P5C synthase
PAT1	Proton-coupled amino acid transporter 1
PC	Proline cycle
PI3K	Phosphoinositide 3-kinase
PPARγ	Peroxisome proliferator-activated receptor gamma
PRPP	Phosphoribosyl pyrophosphate
Pro	Proline
PRODH/POX	Proline dehydrogenase/oxidase
PROT	L-proline transporter PROT
PS	Proline shuttle
PYCR1/2/L	P5C reductase 1/2/L
R-5-P	Ribose-5-phosphate
ROS	Reactive oxygen species
Ru-5-P	Ribulose-5-phosphate
SIT1	Sodium/imino-acid transporter 1
SNAT1/2	Sodium-coupled neutral amino acid transporter 1/2
STAT3	Signal transducer and activator of transcription 3
TCA	Tricarboxylic acid
UC	Urea cycle
VEGF	Vascular endothelial growth factor
V_max_	Maximum velocity
α-KG	α-ketoglutarate
γ-GPR	γ-glutamyl phosphate reductase
*δ*OAT	Ornithine δ-aminotransferase
